# Surface Layer Fluorination-Modulated Space Charge Behaviors in HVDC Cable Accessory

**DOI:** 10.3390/polym10050500

**Published:** 2018-05-04

**Authors:** Jin Li, Boxue Du, Jingang Su, Hucheng Liang, Yong Liu

**Affiliations:** Key Laboratory of Smart Grid of Ministry of Education, School of Electrical and Information Engineering, Tianjin University, Tianjin 300072, China; duboxue@tju.edu.cn (B.D.); sujg1357@126.com (J.S.); hcliang@tju.edu.cn (H.L.); tjuliuyong@tju.edu.cn (Y.L.)

**Keywords:** HVDC cable accessory, space charge, direct fluorination, trap distribution

## Abstract

Space charges tend to accumulate on the surface and at the interface of ethylene–propylene–diene terpolymer (EPDM), serving as high voltage direct current (HVDC) cable accessory insulation, which likely induces electrical field distortion and dielectric breakdown. Direct fluorination is an effective method to modify the surface characteristics of the EPDM without altering the bulk properties too much. In this paper, the surface morphology, hydrophobic properties, relative permittivity, and DC conductivity of the EPDM before and after fluorination treatment were tested. Furthermore, the surface and interface charge behaviors in the HVDC cable accessory were investigated by the pulsed electroacoustic (PEA) method, and explained from the point of view of trap distribution. The results show that fluorination helps the EPDM polymer obtain lower surface energy and relative permittivity, which is beneficial to the interface match in composite insulation systems. The lowest degree of space charge accumulation occurs in EPDM with 30 min of fluorination. After analyzing the results of the 3D potentials and the density of states (DOS) behaviors in EPDM before and after fluorination, it can be found that fluorination treatment introduces shallower electron traps, and the special electrostatic potential after fluorination can significantly suppress the space charge accumulation at the interface in the HVDC cable accessory.

## 1. Introduction

Polymer dielectrics are always preferred in the HVDC power cable insulation system, considering their excellent mechanical and electrical properties [1,2,3]. EPDM (ethylene–propylene–diene terpolymer), which performs better than traditional silicone rubber, has been widely applied as the insulation in cable accessories, and decreases the cable joint size and installation cost, which in turn, expands the application of extruded polymer cable in underground and subsea power transmission [4]. However, space charges are inclined to accumulate at the interface in the composite insulation system consisting of different dielectrics, like the HVDC cable accessory, which will then distort the partial electrical field, cause discharge along the interface, and induce dielectric breakdown in the HVDC cable system [5,6]. Vu et al. considered that the interface charge was produced by the Maxwell–Wagner polarization in the bilayer insulation [7]. There have also been many papers reporting that the surface state and interface barrier have strong correlation with the accumulation of the interface charge [8,9]. Therefore, a theoretical model that includes the multi-factors of interface charge behaviors and methods for their effective suppression are urgently needed to support the reliability and safety of the HVDC cable accessory.

Surface modification has been proven as a potential method for optimizing the surface properties without changing the mechanical and electrical behaviors of the entire dielectric [10,11]. Direct fluorination is an effective method of polymer surface modification, which regulates the surface’s mechanical and chemical properties by replacing the active hydrogen atoms with fluorine atoms, and is usually achieved in a gas mixture composited of F_2_ with N_2_ or O_2_ [12]. Du et al. investigated the effects of direct fluorination on the electrical properties of both outdoor and indoor insulators, and revealed that surface fluorination could reduce the accumulation of surface charges and improve the flashover voltage [13,14]. An et al. obtained various types of temperature fluorinated silicone rubber, and found that there were two effects from the substitution reaction and fluorine diffusion during the fluorination process [15]. Most of the related research focuses on the fluorination-dependent surface charge of insulators. However, the effects of direct fluorination on the interface properties, the space charge behaviors in HVDC cable bilayer systems, and related mechanisms, need further investigation. In this paper, different fluorinated low density polyethylene (LDPE) and EPDM bilayered combinations were obtained through direct fluorination with F_2_/N_2_ mixture. The scanning electron microscope (SEM), hydrophobic tests, DC volume conductivity, and dielectric spectrum were employed to analyze the surface chemical and physical changes after surface modification. The surface charge behaviors of the fluorinated EPDM and interface charge characteristics of the EPDM/LDPE bilayer dielectrics were investigated to reveal the interface trap modification achieved by direct fluorination. Results show that 30 min of fluorination can accelerate the surface charge decay through modifying the DC conductivity. Additionally, the interface charge reaches a minimal value with 30 min fluorinated EPDM in these experiments, to which the more ideal dielectric match and shallower interface traps in the bilayer dielectrics are attributed. The experimental investigations present very promising results with an appropriate direct fluorination modification on the cable accessory insulation surface, which is a potential method to improve the insulation match and suppress space charge accumulation in HVDC cable accessory with considerable industrial feasibility.

## 2. Materials and Methods

### 2.1. Sample Preparation

The EPDM matrix in the experiments was commercially available from Mitsui Chemicals (Tokyo, Japan), and had excellent properties of both extrusion and injection. The mixture of EPDM with 1 wt % vulcanization agent (DMDBH) was completed at 433 K in a double roller mill for 15 min, and was heat-pressed at 453 K for 5 min with a vulcanizer under a pressure of 10 MPa. After this, the compound was naturally cooled down to room temperature. The preforming process for LDPE granules was performed under 10 MPa at 403 K in 10 min. The thicknesses of the EPDM and LDPE were 350 and 200 μm, respectively. All samples were wiped off with ethyl alcohol and put into a drying oven at 298 K for 12 h before fluorination. The samples of EPDM and LDPE were fixed in the reaction still without contacting the rampart, in order to create a sufficient reaction. The fluorination of the samples was performed at 298 K in a mixture gas of F_2_ and N_2_ (vol % = 1:4) at 50 kPa for 0, 15, 30, and 60 min, respectively (EPDM0, EPDM15, EPDM30, and EPDM60). The 0 and 30 min LDPE fluorination process were completed under the same conditions (LDPE0 and LDPE30).

### 2.2. Hydrophobic Test

A static contact angle (SCA) measurement system (Fangrui Corp., Shanghai, China) was employed to obtain the hydrophobicity of the EPDM and LDPE samples. Purified water (10 μL) was dropped onto the sample surface. Next, the SCA was acquired using an online goniometer. Ten specimens for each kind of treatment were tested, and then the average SCA was recorded.

### 2.3. Dielectric Property and DC Volume Conductivity Measurement

The dielectric properties of the LDPE and EPDM with different fluorination treatments were obtained by using the Novocontrol Concept 80 (NOVOCONTROL Technologies GmbH & Co. KG, Montabaur, Germany). There was silver colloid between the sample and the electrode to ensure ideal contact. The ambient temperature of the tests was 298 K. Relative permittivity was obtained with a 1 kV voltage at 50 Hz. The DC conductivity under 20 kV/mm was measured by a three-electrode system. To avoid partial discharge during the experiments, a silicone ribbon was laid at the electrode’s periphery. All samples were kept in a vacuum chamber under 333 K for 24 hours before the tests to eliminate water absorption.

### 2.4. Surface and Space Charge Measurement

The surface charge dynamic behaviors of the fluorinated and original EPDM samples were initiated by a 5, 7, and 9 kV corona discharge, and measured using a Kelvin type probe (TRECK, Inc., Cincinnati, OH, USA), whose experimentation with which was introduced in detail in our previous work [16]. The space charge behaviors, including the polarization and depolarization processes of the different fluorinated bilayer samples, were obtained by the pulsed electroacoustic (PEA) method [17]. Each combination of LDPE/EPDM samples was stressed under 20 kV/mm for 30 min, and thereafter, the depolarization process was sustained for 10 min.

## 3. Results

### 3.1. SEM and EDAX Analysis

**Figure 1:**
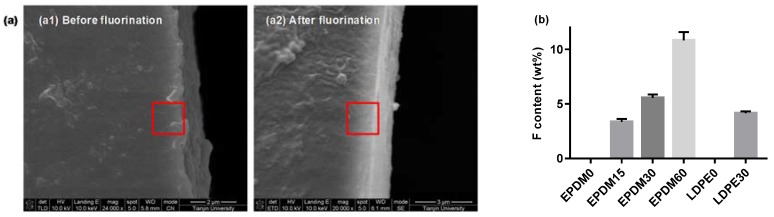
Scanning electron microscope of the ethylene–propylene–diene terpolymer (EPDM) and low density polyethylene (LDPE) cross-section. (**a**) SEM pictures; (**b**) fluorine contents.

The physicochemical characteristics of the polymer surface layers, fluorinated for different lengths of time, were evaluated using SEM and EDAX techniques. Figure 1 shows direct evidence for fluorinated layer formation within the light-colored areas, where the thickness is about 2 μm. By contrast, it is smoother for the surface-treated samples, which may provide a more ideal contact in bilayer dielectrics. Furthermore, energy dispersive analysis was employed to quantitatively evaluate the degree of fluorination. Results show that the fluorine element content in the red frame of the surface layer increases with the treatment time for both the EPDM and LDPE. It is concluded in our previous papers that hydrogen atoms and hydroxyl groups can be replaced by fluorine atoms introduced into the surface layer during the fluorination process, and that fluorine atoms can be added to the carbon–carbon bonds. The crosslinking reactions occur simultaneously.

### 3.2. Hydrophobic Properties

**Figure 2:**
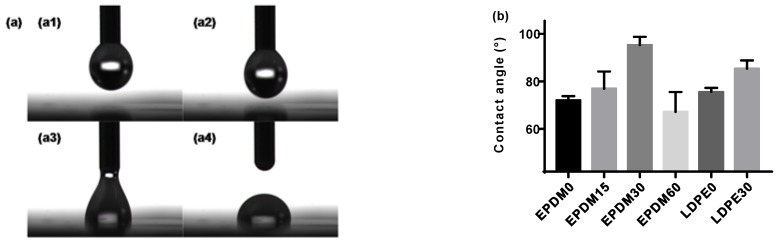
Hydrophobic properties of the fluorinated EPDM and LDPE. (**a**) Hydrophobicity measurement; (**b**) contact angle.

The hydrophobic properties of a material can be defined by the ability of a liquid to accumulate on its solid surface. Measuring or estimating the static contact angle between the solid and the liquid involved, under the action of surface forces, can give an understanding of this property [18]. The process of a droplet of distilled water slowly falling onto the determinand was recorded by a camera, as shown in Figure 2a, followed by production of the contact angles. The results of the contact angle for the EPDM and LDPE samples with different fluorination treatments are shown in Figure 2b. It is observed that the values of the contact angle for both the fluorinated EPDM and LDPE are higher than those of the neat samples. Usually, a few introduced fluorine atoms will decrease the contact angle of polymers, due to the enhanced surface polarity. However, another factor that should be considered is that the surface roughness can also be improved by fluorination, as shown in Figure 1, which will then increase the contact angle. With increasing fluorination time, more –(CF_n_) will be formed, which has a lower surface free energy than –(CH_n_) and helps to improve a material’s hydrophobic properties. Further, the effects of surface crosslinking during the fluorination process lead to larger contact angles [19]. The fact remains that there is a change in the surface tension because of surface roughness changes, crosslinking, and fluoride group formation, except for in the EPDM60, due to chain scission. A smoother surface after fluorination treatment can help to realize an ideal contact between the EPDM and LDPE in HVDC cable accessory. Additionally, the surface energy has a strong relationship with the surface trap distribution. In general, the change of the surface morphology of the fluorinated samples will cause different dielectric behaviors, which we will explore further in later sections of this paper.

### 3.3. Relative Permittivity and DC Conductivity

**Figure 3:**
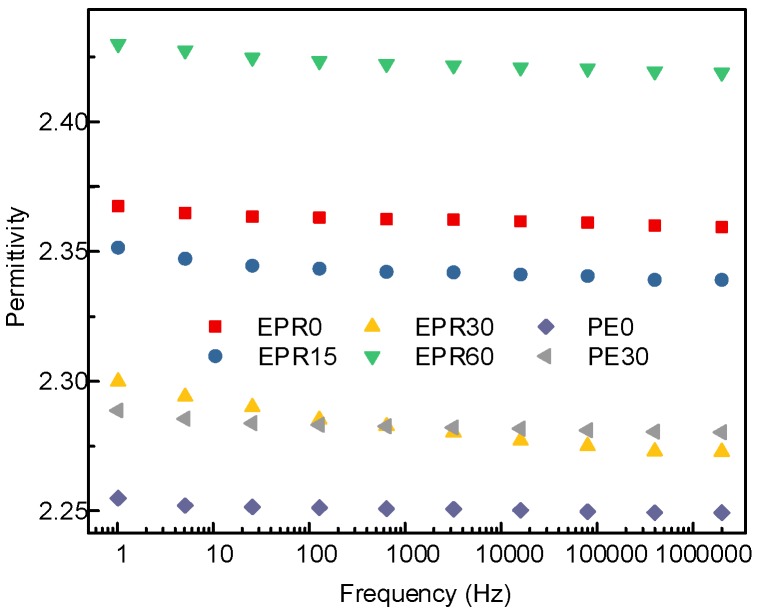
Relative permittivity of different fluorinated samples.

Lowering the relative permittivity of the polymer will make the transient electric field distribution in the composite insulation system more reasonable. The field strength distortion will be relatively reduced, which is more conducive to improving the dielectric strength and reliability of the composite insulation. The relative permittivity of the EPDM and LDPE samples modified by fluorination for different lengths of time was measured by the Novocontrol Concept 80, as shown in Figure 3. It can be seen from the figure that the relative permittivity of the EPDM and LDPE decreases slightly with increasing frequency, which is the dielectric relaxation phenomenon caused by the inertia of the molecule itself, and the viscosity of the polymer chains at a higher frequency. The relative permittivity of the non-fluorinated EPDM samples was between 2.36 and 2.37. When the surface modification time was increased from 0 to 30 min, the relative permittivity of the EPDM decreased from 2.37 to 2.28 at 50 Hz. However, when the surface treatment time increases and reaches 60 min, the relative permittivity of the sample reached 2.42. For the LDPE samples, the relative permittivity at power frequency decreased from 2.27 to 2.24, with the modification of the surface molecular structure for 30 min.

The experimental results show that a certain degree of surface modification can reduce the dielectric constant, which is beneficial for the electrical field distribution in the HVDC cable accessory, and can be attributed to the special atomic structure and polarization characteristics of the fluoride groups. The mechanisms that achieve this have three main aspects: the dipolar polarization, crosslinking, and free volume. With increasing fluorination time, the –(CH_n_) groups are gradually substituted by –(CF_n_) in the order of tertiary hydrogen, secondary hydrogen, then primary hydrogen. Therefore, the large scale of the appearance of the dipolar polarization in the –(CF_n_) groups will play a decisive role in the polarization process instead of the electronic polarization, the former of which ends with lower relative permittivity [20,21,22]. In addition, due to the possibility of crosslinking of the free radicals in the molecular chain during fluorination, the surface molecules of the sample undergo crosslinking to further restrict the orientation polarization of the polymer molecules. Moreover, the substitution of hydrogen atoms by fluorine atoms will increase the free volume of the rubbery polymer, reduce the number of molecules per unit volume, and thus, reduce the polarizability and relative dielectric constant of the polymer. However, excessive fluorination will destroy the regularity of the polymer matrix, resulting in new molecular chain scission, small polar molecules, and other polar groups, which in turn, increases the degree of polarization.

**Figure 4:**
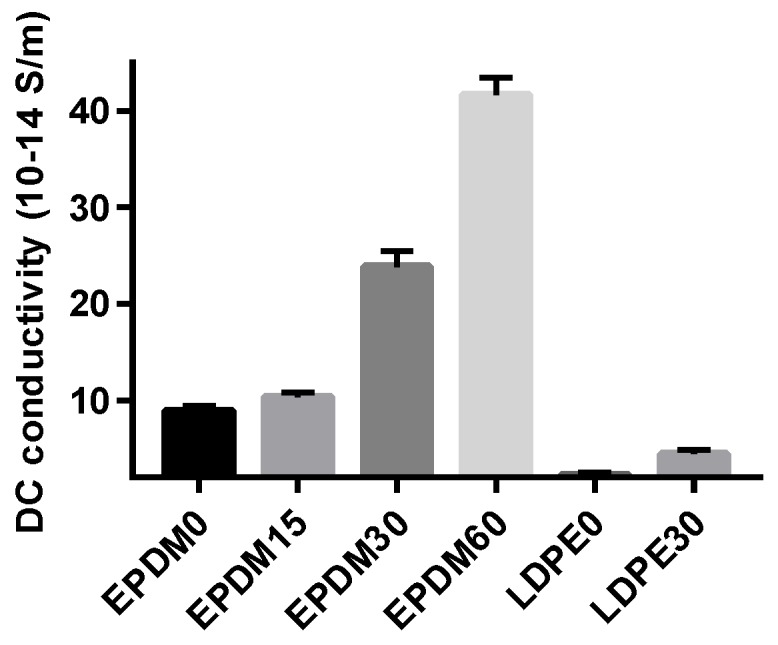
DC conductivity time of different fluorinated samples under 20 kV/mm.

The conductance current can reflect many microscopic characteristics of carrier transport in the dielectrics, such as carrier injection, charge trapping and detrapping, trap carrier density, and the electric aging threshold, which are widely used in research of the semiconductor and dielectric materials charge transport process [23]. In this paper, the DC conductivity based on the final value of the conductance current was employed to reflect the effects of surface modification, as shown in Figure 4. The conductivity of the EPDM samples is generally higher than that of the LDPE samples, as the energy gap of EPDM (~6–7 eV) is much lower than that of LDPE (~10 eV). In summary, the conductivity of both the EPDM and LDPE samples measured at 20 kV/mm increases with fluoridation time. This is because the modification of the surface molecules causes the polymer surface to form a fluorinated layer consisting of –(CF_n_), which has a larger conductance [24], resulting in an increase in the conductivity of the fluorinated EPDM and LDPE. At the other extreme, though the conductance is elevated by direct fluorination, the space charge is not inclined to accumulate at the interface or on the surface of the insulation system, which is a more serious threat to the safety of the HVDC cable operation.

### 3.4. Surface Potential Decay and Surface Trap Distribution

**Figure 5:**
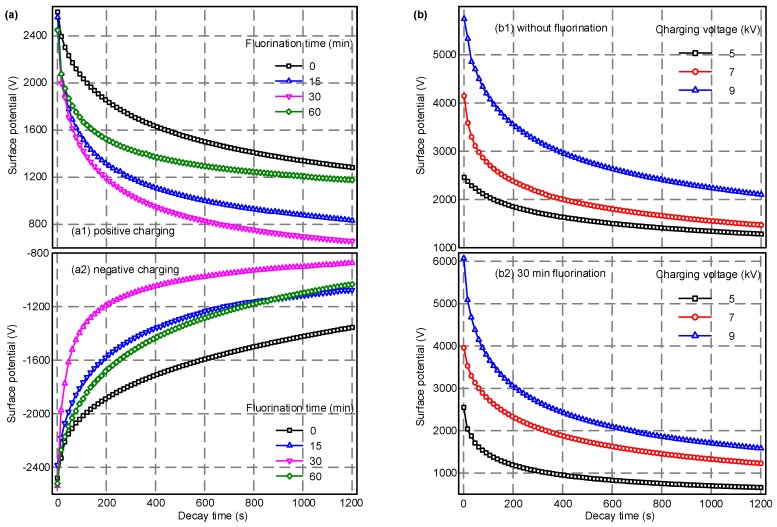
The surface potential decay process of samples with various fluorination time under different charging voltage levels. (**a**) Effects of fluorination time; (**b**) effects of charging voltage.

Figure 5a shows the relationship between the surface potential of EPDM and the dissipation time under 5 kV corona voltage with different lengths of fluorination time. As seen from the curves, as the surface modification time increases from 0 to 60 min, the positive and negative initial surface potentials of the samples do not change much. The surface potential decays rapidly in the initial stage and reaches a stationary phase after about twenty minutes. Regardless of the corona polarity, as the fluorination time increases from 0 to 30 min, the decay rate of the surface potential gradually increases, and the time required to reach a steady value becomes shorter. However, when the fluorination time was further increased to 60 min, the decay rate of the surface potential was reduced, and the surface potential value at the end of the test was increased when compared to the 30 min fluorination treatment. This is because the crosslinking effects caused by excessive fluorination have a negative effect on the migration of surface charges. A moderate modification of the surface molecular structure can form a –(CF)_n_ layer on the surface of the material, which will accelerate the charge migration and dissipation process. Figure 5b illustrates the effects of different corona voltages on the EPDM surface potential dynamic behaviors. It can be seen from the figure that for the non-fluorinated samples, the initial surface potential increases from 2450 to 5750 V as the corona voltage increases from 5 to 9 kV. For the 30 min fluorinated samples, the initial surface potential increases from 2490 to 6050 V. With the increase of the corona voltage level, the initial surface potential continues to increase while the surface potential decay rate also increases, and its final value is higher. The above result indicates that a more intense corona, achieved by a higher voltage level, induces more charges to become trapped on the polymer surface and, at the same time, the inside electric field is increased due to more surface charge accumulation. This also enhances the motivation for surface charge transport to occur, and ends in a more rapid dissipation procedure.

**Figure 6:**
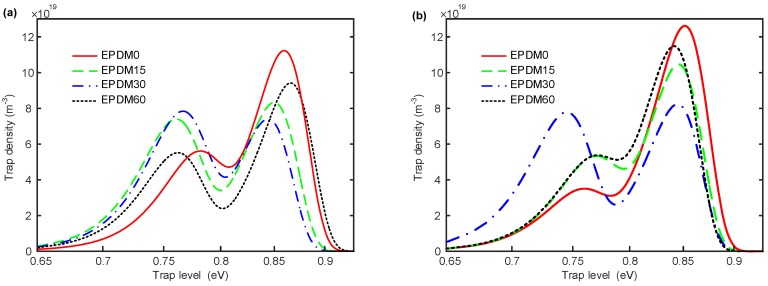
Surface trap distribution of samples with various fluorination time under 5 kV corona voltage. (**a**) Hole traps; (**b**) electron traps.

The surface trap distribution is calculated and summarized to analyze the effects of direct fluorination on the surface charge behaviors, whose calculation process is presented in Figure S1 (Supplementary Information). The results of the surface trap distribution, shown in Figure 6a,b, are similar to each other. There are two peaks of trap density—deep traps and shallow traps. The deep and shallow traps are relative. It is easy for the carriers in shallow traps to detrap, while it is harder to detrap from deep traps, which means deep traps have a higher probability of capturing space charges. The density of both deep electron and hole traps in neat EPDM are much larger than the shallow traps, indicating that mainly deep traps exist in EPDM, and these can be expected to capture charges on the sample surface and at the interface. Furthermore, the density of deep electron traps is higher than that of the hole traps, whereas the shallow electron traps have a lower density when compared to the hole traps. The energy distribution of hole traps in the EPDM implies that the hole traps are easier to modify by fluorination, but do not change much, while for the electron trap distribution, only 30 min of fluorination significantly affects the shallow trap density. In general, direct fluorination increases the proportion of the shallow traps for both the hole and electron traps, and decreases the density of deep traps.

### 3.5. Interface Charge Behaviors and Interface Trap Distribution

**Figure 7:**
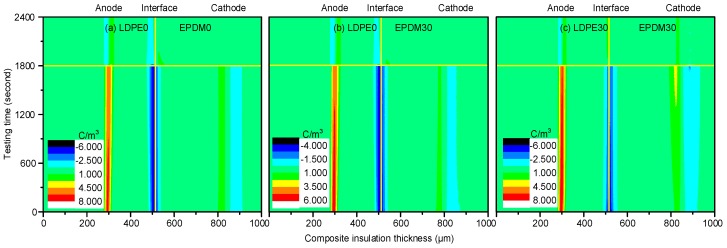
Space charge distribution during the polarization and depolarization processes. (**a**) LDPE0/EPDM0; (**b**) LDPE0/EPDM30; and (**c**) LDPE30/EPDM30.

Figure 7 illustrates the space charge distribution of the LDPE/EPDM composite insulation. The interface charge polarity is negative, as it is when the voltage is applied to the EPDM. In Figure 7a, a large number of charges accumulate at the interface with a density of 7.55 C/m^3^, due to 1800 s of stressing. Figure 7b shows the dynamic space charge behaviors of the 30 min fluorinated EPDM composed bilayer insulation system. Compared with the non-fluorinated EPDM samples, the interfacial charge density is decreased to 3.65 C/m^3^. This level of fluorine produces sufficient inhibition of the interface charge accumulation. Figure 7c demonstrates the depolarization and polarization processes of both of the fluorinated samples (LDPE30/EPDM30). For this group of samples, the interface charge density first increases and then decreases, which is caused by the slow release of the quick polarized dipoles in the fluorinated polymers. At the same time, more heterogeneous charge is injected from the cathode.

**Figure 8:**
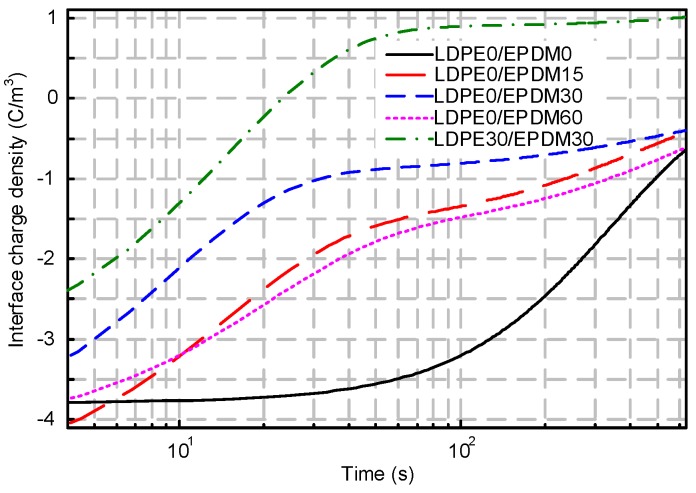
Interface charge dissipation for different sample arrangements.

Figure 8 shows the interfacial charge dissipation of the EPDM/LDPE bilayer system after stressing for 30 min. With the passage of time, the space charges at the interface are divided into positive and negative charges which migrate to the anode and cathode, respectively, under the electric field formed by residual charges. In the neat samples, the space charge in the LDPE dissipates faster at the beginning, while the charges in EPDM appear to retain remnants, despite the depolarization time. A similar trend can also be found during interfacial charge diffusion for the simply fluorinated EPDM bilayer system. Without regard to the initial charge density of the fluorinated samples, the dissipation speed is much higher than in the original samples. It is interesting to note that the polarity of the interface charge changes gradually from negative to positive for the LDPE30/EPDM30 bilayer system. It may be caused by the released polarization charges from the fluorinated layers of both the LDPE and EPDM.

The results for the deep and shallow traps of the bilayer dielectrics with individual EPDM fluorination calculated according to the Table 1 and Table S1 (Supplementary Information). It can be seen that as the fluorination time increases from 0 to 30 min, the shallow trap energy level decreases from 0.95 to 0.92 eV, whereas the deep trap level increases from 1.03 to 1.10 eV. When the fluorination time ranges from 30 to 60 min, the shallow trap level also rises from 0.92 to 0.93 eV, while the deep trap level drops from 1.10 to 1.08 eV. The charge dissipation in the initial stage of depolarization is mainly due to the charge detrapping from the shallow traps, which occurs relatively rapidly. When the charges in the shallow traps are completely detached, the charge detrapping process in the deep traps becomes more difficult. Therefore, the interface charge dissipation rate decreases. The trap energy level distribution is relatively narrow in the original sample, and the charge dissipation tendency is relatively flat. The average barrier to be overcome for the interfacial charge detrapping of the fluorinated sample is relatively lower, so the dissipation speed of the charge in the initial phase is relatively fast.

### 3.6. 3D Potential and DOS Behaviors

**Figure 9:**
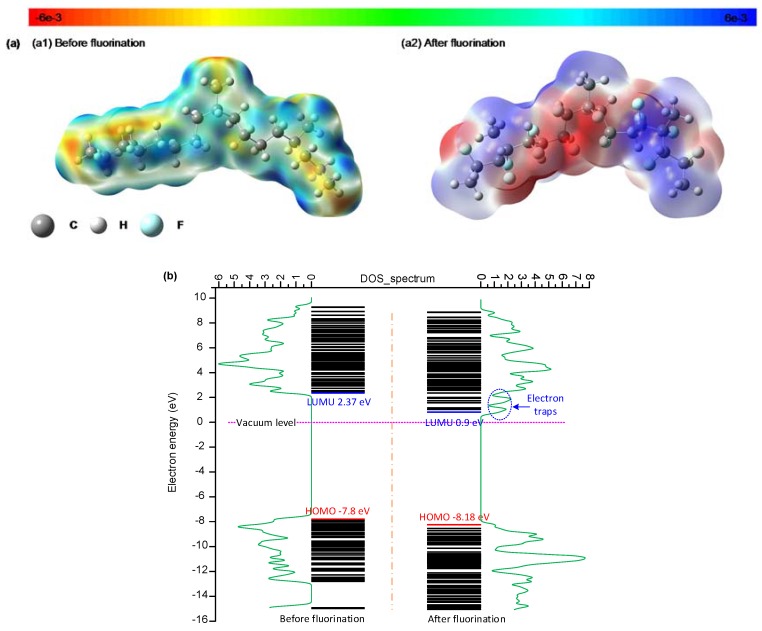
(**a**) 3D potential distribution of the EPDM surface layer before and after fluorination; (**b**) density of states (DOS) and energy level of the EPDM surface layer before and after fluorination.

According to the above results, the space charge accumulation on the sample surface and at the interface is significantly suppressed in the 30 min fluorinated EPDM samples, due to the increased proportion of shallow traps. It is believed that the trap distribution in the polymer surface layer is significantly correlated with the electrostatic potential upon fluorination treatment. Figure 9a shows the 3D electrostatic potential distribution in the EPDM surface layer before and after fluorination. The third monomer, ethylidene norbornene (ENB), is not included in the EPDM model, given its very low contents in the unit molecular chain (4.6%). In the 3D electrostatic potential distribution, the warm color shows the negatively charged region, and the cold color shows the positively charged region. As the electronegativity values between the carbon of the main chain and the hydrogen of the side chain are different, the orbital electron is biased in the EPDM. The carbon of the main chain and the hydrogen of the side chain are respectively negatively and positively charged in the EPDM, as shown in Figure 9(a1). For the EPDM samples after fluorination, the colors of both the warm and cold regions deepen, which means that the electrons and holes are easily captured by the cage skeleton.

Figure 9b shows the electronic energy level and DOS (density of states) spectrum in the EPDM surface layer before and after fluorination. In the neat EPDM surface, the lowest unoccupied molecular orbital (LUMU) and the highest occupied molecular orbital (HOMO) are 2.37 eV and −7.8 eV respectively, and consequently, the energy gap is 10.17 eV. After the fluorination treatment, the LUMU and HOMO are 0.9 and −8.18 eV instead, and the energy gap is 9.08 eV. Therefore, it is easy for the electrons to jump over the narrow energy gap and join the conduction process, which is consistent with the results of increased DC conductivity after fluorination. According to the DOS spectrum, fluorination can generate new electron traps in the EPDM surface as the LUMU in the fluorinated samples is lower than in the neat samples. There are two electron traps after treatment, which are introduced from the formation of –(CF_n_). The newly generated electron traps from fluorination are mainly shallow traps, which result in less trapped charges on the EPDM surface and at the interface of the EPDM/LDPE composite system, according to the above test results. It should be pointed out that the electronic energy level and DOS spectrum results are based on ideal molecular groups being used to characterize the effects of fluorine atom replacement, which ignores factors such as hanging bonds, dislocation, and so forth.

## 4. Discussion

It is widely accepted that the study of space charge accumulation in a composite insulating system needs to consider the effects of surface state, as the surface characteristics and electronic structure are significantly different to the interior parts of a dielectric. The periodic potential fields of solids are abruptly interrupted and distorted at the surface. In order to maintain the stability of the system, the atomic arrangement must be adjusted, which results in many additional electronic states; namely, the surface states [25]. The presence of surface states will lead to an increase in trap density and a deeper distribution of energy levels in the surface layer of the dielectric, which results in the accumulation of injected charge near the interface [26]. The surface molecular structure can be modified and redesigned by the direct fluorination method to modify the effects of the surface state on space charge accumulation. Though the trap characteristics have some differences in terms of the trap energy level measured by different methods, the distribution trends are similar for the surface traps and interface traps. Due to the molecular structure modification of the surface layer by atom substitution, the binding effects of the surface state on the space charge accumulation on the surface and at the interface have been substantially suppressed by increasing the proportion of the shallow traps, which also improves the match extent of the interface in the HVDC cable accessory [27,28].

## 5. Conclusions

The improvement of the dielectric properties of EPDM after direct fluorination treatment was analyzed. SEM and EDAX results have proven that fluorinated EPDM samples have a surface layer containing fluorine atoms which are formed on the smoother surface. Static contact angle tests have shown stronger hydrophobicity and lower surface energy for these samples, even with transitory treatments. Experiments performed under DC electrical fields suggest that direct fluorination can increase the volume conductivity, and thereby accelerate surface charge dissipation. The dielectric spectrum upon a wide range of frequencies reveals that the fluorination less than 30 min causes a decrease in the permittivity of EPDM, which is beneficial to the dielectric match in HVDC cable accessory. Less space charges are accumulated on the surface and at the interface, which is attributed to the effects of more shallow traps introduced by direct fluorination, and proven by the 3D potential and DOS behaviors. Conversely, excessive fluorination causes degradation of the chemical groups, which forms defects, and thus promotes the generation of deep traps. The results derived from this paper are of great significance to the improvement of the electrical properties of HVDC cable accessory insulation, thus promoting its long-term reliability.

## Figures and Tables

**Table 1:** Relationship between apparent interface trap depth and surface fluorination treatment.

Sample Arrangement	∆min (eV)	∆max (eV)
LDPE0/EPDM0	0.95	1.03
LDPE0/EPDM15	0.93	1.07
LDPE0/EPDM30	0.92	1.10
LDPE0/EPDM60	0.93	1.08

## Supplementary Materials

The following are available online at http://www.mdpi.com/2073-4360/10/5/500/s1, Figure S1, Table S1.
