# Supplementary material for: Surface Layer Fluorination-Modulated Space Charge Behaviors in HVDC Cable Accessory
Source: Polymers (Basel). 2018 May 4;10(5):500. doi: 10.3390/polym10050500 (PMC6415413; doi:10.3390/polym10050500)
Supplement: Supplementary file 1 [file polymers-10-00500-s001.zip › polymers-294054-supplementary.pdf]

Supplementary Materials for

Surface Layer Fluorination Modulated Space Charge Behaviors in HVDC Cable Accessory

Jin Li *, Boxue Du, Jingang Su, Hucheng Liang and Yong Liu

Key Laboratory of Smart Grid of Ministry of Education, School of Electrical and Information Engineering, Tianjin University, Tianjin 300072, China; duboxue@tju.edu.cn (B. X. Du); sujg1357@126.com (J. G. Su); hcliang@tju.edu.cn (H. C. Liang); tjuliuyong@tju.edu.cn (Y. Liu)

* Correspondence: lijin@tju.edu.cn; Tel.: +86-159-221-80552

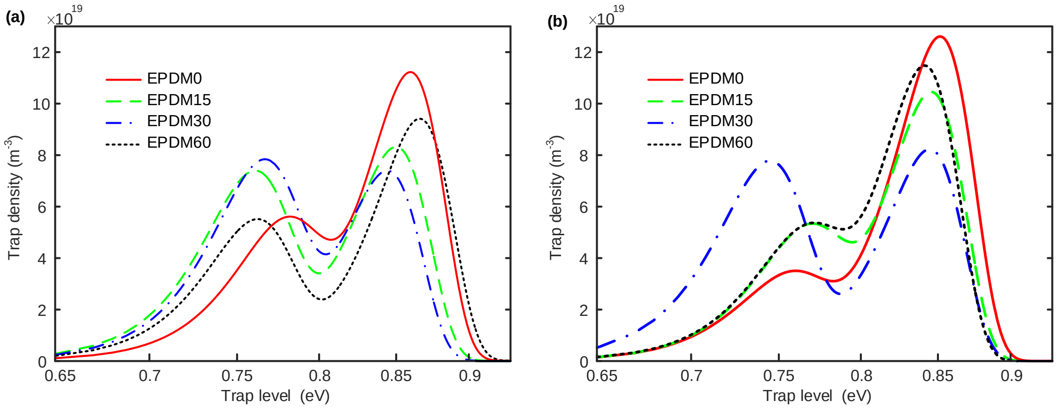

Figure S1. Surface trap distribution of samples with various fluorination time under 5 kV corona voltage. (a) hole traps, (b) electron traps

There are a large number of trap levels in the forbidden band of polymer materials, which are caused by the molecular branches, the interface between amorphous and crystalline regions, impurities introduced during the preparation process, various structural defects and so on. The trap level changes will directly affect the carriers trapping, detrapping, migration, recombination and other processes, so we can infer the microscopic changes of the dielectric by analyzing the trap level distribution of the material [1]. The surface potential decay (SPD) method is considered as a useful technique to evaluate the trap distribution of dielectrics. Usually, the $t\mathrm{d}V/\mathrm{d}t$ with the time of the captured charge to escape from the traps are employed to characterize the energy level and density of the surface state. The relationship between the trap density (*N*t) and the energy level (*E*t) can be expressed as bellow:

$$N_t = \frac{\varepsilon_0 \cdot \varepsilon_r}{q_e \cdot L} \cdot t \frac{dV}{dt} \tag{1}$$

$$E_t = kT \ln\left(v \cdot t\right) \tag{2}$$

Where ε_0, ε_r are the vacuum dielectric constant and relative dielectric constant, q_e is the coulomb's quantity of electron, *L* is the thickness of the sample, *V* is the surface potential, *t* is the decay time of the surface potential, *K* the Boltzmann's constant, *T* is the temperature, υ is the attempt to escape frequency.

Table S1. Relationship between apparent interface trap depth and surface fluorination treatment

Sample arrangement	Δmin (eV)	Δmax (eV)
LDPE0/EPDM0	0.95	1.03
LDPE0/EPDM15	0.93	1.07
LDPE0/EPDM30	0.92	1.10
LDPE0/EPDM60	0.93	1.08

Further analysis of the interface charge dissipation behaviors will be performed from the view of carrier de-trapping process. The apparent trap depth can be calculated from the results of the interface charge dissipation by Dissado [2]. Suppose that the charges are captured by the traps distributing from Δmax to Δmin, and then the boundary values can be obtained from the time dependent interface charge dissipation process,

$$\begin{aligned} \Delta_{\min} &= kT \ln(t_1 v) \\ \Delta_{\max} &= kT \ln(t_2 v) \end{aligned} \tag{1}$$

where $v=KT/h$ is the de-trapping attempt frequency, K the Boltzmann constant, h the Planck constant and T the temperature. The shallowest traps start to be emptied at t_1 and the deepest traps start to be emptied at time t_2.

---

1 Xie, Q.; Lin, H.; Zhang, S.; Wang, R.; Kong, F.; Shao, T. Deposition of SiCxHyOz thin film on epoxy resin by nanosecond pulsed APPJ for improving the surface insulating performance. *Plasma Sci. Tech.*, **2018**, 20, pp. 025504. DOI: 10.1088/2058-6272/aa97d0.

2 Dissado, L. A.; Griseri, V.; Peasgood, W.; Cooper, E. S. Decay of space charge in a glassy epoxy resin following voltage removal. *IEEE Trans. Dielectr. Electr. Insul.*, **2006**, 13, pp. 903-916. DOI: 10.1109/TDEI.2006.1667752.
